# Validity of a Minimally Invasive Autopsy for Cause of Death Determination in Adults in Mozambique: An Observational Study

**DOI:** 10.1371/journal.pmed.1002171

**Published:** 2016-11-22

**Authors:** Paola Castillo, Miguel J. Martínez, Esperança Ussene, Dercio Jordao, Lucilia Lovane, Mamudo R. Ismail, Carla Carrilho, Cesaltina Lorenzoni, Fabiola Fernandes, Rosa Bene, Antonio Palhares, Luiz Ferreira, Marcus Lacerda, Inacio Mandomando, Jordi Vila, Juan Carlos Hurtado, Khátia Munguambe, Maria Maixenchs, Ariadna Sanz, Llorenç Quintó, Eusebio Macete, Pedro Alonso, Quique Bassat, Clara Menéndez, Jaume Ordi

**Affiliations:** 1 ISGlobal, Barcelona Centre for International Health Research (CRESIB), Hospital Clinic of Barcelona, Universitat de Barcelona, Barcelona, Spain; 2 Department of Microbiology, Hospital Clinic of Barcelona, Universitat de Barcelona, Barcelona, Spain; 3 Department of Pathology, Maputo Central Hospital, Maputo, Mozambique; 4 Faculty of Medicine, Eduardo Mondlane University, Maputo, Mozambique; 5 Department of Medicine, Maputo Central Hospital, Maputo, Mozambique; 6 Fundação de Medicina Tropical Doutor Heitor Viera Dourado, Manaus, Amazonas, Brazil; 7 Centro de Investigação em Saúde de Manhiça, Maputo, Mozambique; 8 Catalan Institution for Research and Advanced Studies (ICREA), Barcelona, Spain; 9 Department of Pathology, Hospital Clinic of Barcelona, Universitat de Barcelona, Barcelona, Spain; Umeå Centre for Global Health Research, Umeå University, SWEDEN

## Abstract

**Background:**

There is an urgent need to identify tools able to provide reliable information on the cause of death in low-income regions, since current methods (verbal autopsy, clinical records, and complete autopsies) are either inaccurate, not feasible, or poorly accepted. We aimed to compare the performance of a standardized minimally invasive autopsy (MIA) approach with that of the gold standard, the complete diagnostic autopsy (CDA), in a series of adults who died at Maputo Central Hospital in Mozambique.

**Methods and Findings:**

In this observational study, coupled MIAs and CDAs were performed in 112 deceased patients. The MIA analyses were done blindly, without knowledge of the clinical data or the results of the CDA. We compared the MIA diagnosis with the CDA diagnosis of cause of death.

CDA diagnoses comprised infectious diseases (80; 71.4%), malignant tumors (16; 14.3%), and other diseases, including non-infectious cardiovascular, gastrointestinal, kidney, and lung diseases (16; 14.3%). A MIA diagnosis was obtained in 100/112 (89.2%) cases. The overall concordance between the MIA diagnosis and CDA diagnosis was 75.9% (85/112). The concordance was higher for infectious diseases and malignant tumors (63/80 [78.8%] and 13/16 [81.3%], respectively) than for other diseases (9/16; 56.2%). The specific microorganisms causing death were identified in the MIA in 62/74 (83.8%) of the infectious disease deaths with a recognized cause.

The main limitation of the analysis is that both the MIA and the CDA include some degree of expert subjective interpretation.

**Conclusions:**

A simple MIA procedure can identify the cause of death in many adult deaths in Mozambique. This tool could have a major role in improving the understanding and surveillance of causes of death in areas where infectious diseases are a common cause of mortality.

## Abstract 2

Jaume Ordi and colleagues examine the validity of a standardized minimally invasive autopsy approach by comparison with that of the complete diagnostic autopsy in a series of adults who died at Maputo Central Hospital in Mozambique.

## Author Summary

**Why Was This Study Done?:**

- While complete autopsies are considered the gold standard for the determination of cause of death, they are poorly accepted and difficult to perform in middle- and low-income countries. More feasible options, such as verbal autopsies and clinical records, are highly inaccurate.
- Minimally invasive autopsy techniques have been proposed as a more acceptable alternative to complete autopsy, and the results of these techniques have been shown to be relatively accurate; however, current minimally invasive autopsy protocols generally involve high-tech imaging procedures not available in middle- and low-income settings.
- Our study was designed to develop a simplified minimally invasive autopsy method that would be feasible in middle- and low-income countries, and to validate this method against the gold standard.

**What Did the Researchers Do and Find?:**

- The minimally invasive autopsy developed in this study consists of the collection of blood and cerebrospinal fluid samples and the collection of tissue samples from solid organs using biopsy needles, followed by histological and microbiological analyses.
- Coupled minimally invasive and complete autopsies were performed in 112 deceased patients. The samples obtained by both techniques were evaluated independently.
- Concordance between the putative diagnosis obtained with the minimally invasive autopsy and the gold standard diagnosis obtained by complete autopsy was 75.9%.
- The agreement was particularly high for infectious diseases. Moreover, the specific microorganisms causing death were accurately identified in the minimally invasive autopsy samples.

**What Do These Findings Mean?:**

- Minimally invasive autopsy is a reliable tool that can improve our understanding of the causes of death in Mozambique, where infectious diseases are a common cause of mortality.
- The use of this tool could improve health planning and priority setting for the most vulnerable populations in the world.

## Introduction

Current estimates of major causes of mortality in middle- and low-income countries are hampered by the lack of direct and reliable data. Complete diagnostic autopsy (CDA), the gold standard method to determine the cause of death [1], is seldom performed in these countries due to limited human resources and cultural and/or religious backgrounds that negatively influence acceptance and consent in some regions [2,3]. In addition, many deaths occur outside the health system, which precludes not only postmortem evaluation but also frequently the basic medical assistance that allows certification of the death event. Verbal autopsy is a structured interview administered to relatives of the deceased individual and is currently recommended by WHO as an alternative to CDA to overcome this problem in low- and middle-income countries [4–7]. However, although verbal autopsy provides a broad syndromic approach, its performance for etiological diagnosis is very limited, and it tends to misclassify a substantial number of deaths [1]. Finally, clinical records generally show a high rate (10%–30%) of discordance with the results of CDAs [8,9], and this discordance further increases in resource-constrained settings, where the availability of ancillary diagnostic tests such as imaging or microbiological exams is scarce or suboptimal [10,11].

We hypothesized that a simple minimally invasive postmortem sampling procedure could provide reliable etiological information for cause of death investigation and potentially replace other more invasive and less acceptable methods. Recently, we reported the methodology of a standardized minimally invasive autopsy (MIA) [12,13]. This technique involves organ-directed sampling using biopsy needles and provides key fluids and tissue material for histological and microbiological analyses. The procedure is simple and could be easily conducted by trained technicians. In this study, we aimed to analyze the validity of the MIA to determine the cause of death in a series of in-hospital adult deaths in Mozambique, by comparing the MIA diagnosis with the gold standard CDA diagnosis obtained by the same group of experts.

## Methods

### Study Setting and Design

This study received the approval of the following regulatory bodies: the Internal Scientific Committee of the Barcelona Centre for International Health Research (Spain; approved, 6 September 2012), Clinical Research Ethics Committee of the Hospital Clinic of Barcelona (Spain; approved, File 2013/8677), Internal Scientific Committee of the Centro de Investigação em Saúde de Manhiça (Mozambique; approved, Ref. CCI/31/Fev 2013), the Service of Pathology of Maputo Central Hospital (Mozambique; approved, 5 August 2013), and the National Bioethics Committee of Mozambique (Mozambique; approved, Ref. 342/CNBS/13).

This observational study was carried out at the Department of Pathology of the Maputo Central Hospital, a 1,500-bed government-funded quaternary health care center. From November 2013 to March 2015, we conducted up to two coupled MIAs and CDAs per day when cases fulfilled the inclusion criteria. All the patients included in this analysis fulfilled the following criteria: (1) a CDA requested by the clinician as part of the medical evaluation of the patient and (2) informed consent to perform the autopsy given by the relatives. The following exclusion criterion was established: death of traumatic origin. The current paper will present the analyses for adults (patients older than 15 y) excluding maternal deaths. In order to select only two cases per day from among the daily CDA requests received at the department (between 5 and 12 per day) without introducing selection biases, the two patients with death recorded before and closest to the time of 8:00 a.m. were included in the study.

In all cases, informed consent to perform the autopsy was obtained from the relatives of the deceased patients. The STROBE checklist and the prospective analysis plan are included as S1 Text and S2 Text, respectively.

### Autopsy Procedures

The autopsy procedure was performed by a pathologist assisted by a technician. The detailed MIA pathological and microbiological methods have been reported elsewhere [12,13]. In brief, the procedure includes disinfection of the surface of the body, collection of 20 ml each of blood and cerebrospinal fluid (CSF), and puncture of solid organs (liver, lungs, bone marrow, and central nervous system [CNS]) using biopsy needles (14G–16G) to collect samples for microbiological and histological analysis. In addition, the heart, spleen, and kidneys were sampled for histological analysis only.

Immediately after the MIA, the CDA procedure was conducted by a second pathologist not involved with the MIA. The CDA was completed in all cases within 1 h after completion of the MIA. Briefly, a dissection was performed with macroscopic evaluation of all the organs following a standardized macroscopic protocol [14]. In this procedure, samples from the same viscera sampled in the MIA and from any grossly identified lesions were collected for histological and microbiological analysis. The microbiological results of the blood and CSF were also included in the CDA evaluation.

In all cases, both the MIA and the CDA were performed within 24 h after death.

### Histological and Microbiological Analyses

All paraffin blocks and the microbiological samples were sent to the central lab (Hospital Clinic of Barcelona), where two pathologists and a microbiologist, who were not aware of any clinical information or the findings of the CDA, analyzed the histological slides and the microbiological samples from the MIA. After a washout period (minimum 3 mo, range 3–6 mo), the same experts evaluated the slides and microbiological samples obtained at the CDA, while blinded to the findings of the MIA.

All samples collected for histology were routinely stained with hematoxylin and eosin. Ancillary histochemical (e.g., Ziehl-Neelsen) and/or immunohistochemical stains (e.g., *Toxoplasma gondii*) were used, if required, to achieve the diagnosis. The microbiological analyses have been reported in detail [13]. In brief, universal screening was performed for all cases, which included detection of *Plasmodium falciparum* by PCR, detection of antibodies against HIV-1/2, and bacterial/fungal cultures of blood, CSF, liver, lungs, and CNS. In samples positive for antibodies against HIV, the viral load was determined. We routinely applied an additional microbiological screening in all HIV-positive cases, which included real-time PCR in CSF and CNS samples for *T*. *gondii*, *Mycobacterium tuberculosis*, and *Cryptococcus* spp. and real-time PCR in lung samples for *Pneumocystis jirovecii*, *Cryptococcus* spp., and *M*. *tuberculosis*. Other microorganisms were also tested depending on the pathological findings observed in the MIA-obtained tissues.

The samples from the CDAs were analyzed following the same strategy used for the analysis of the MIA samples. The team was aware of all the findings of the CDA (macroscopic, histological, and microbiological results) and of the clinical information.

All the histological and microbiological analyses were performed at the central laboratory in Barcelona, except for blood, CSF, and tissue cultures and HIV analyses, which were done locally (Maputo and Manhiça).

Two scales were developed to grade the strength of the evidence of the autopsy findings, one based on the severity of the pathological findings and the other on the distribution and type of the microorganisms identified (Table 1).

**Table 1:** Strength of the evidence of the autopsy findings identified in the complete diagnostic autopsy and the minimally invasive autopsy.

Level	Evidence	Pathological Findings^a^	Microbiological Findings
0	None	No pathological findings or nonspecific changes	No microorganisms identified
1	Slight	Mild pathological findings, unlikely to be the cause of death	Microorganisms that are frequent contaminants
2	Fair	Mild pathological findings, possibly causing death^b^	Microorganisms that can either represent true pathogens or colonizing/contaminants; mixed infections^c^
3	Moderate	Pathological findings of moderate intensity, probably causing death^b^	Microorganisms that can either represent true pathogens or colonizing/contaminants detected by both molecular and culture-based methods
4	Strong	Severe pathological findings likely to be the cause of death	Microorganisms that represent true pathogens and/or microorganisms consistently detected in ≥4 samples

Microbiology examples according to strength of evidence classification: (1) coagulase-negative staphylococci, group viridans streptococci; (2 and 3) enterobacteriaceae such as *Klebsiella pneumoniae* or *Escherichia coli*, non-fermentative Gram-negative bacilli; (4) *Cryptococcus* spp., *T*. *gondii*, *M*. *tuberculosis*, *P*. *jirovecii*, *Legionella pneumophila*.

^a^Pathological findings include only microscopic changes in the minimally invasive autopsy and both macro- and microscopic changes in the complete diagnostic autopsy.

^b^The finding in the histological (histochemistry, immunohistochemistry) exam of a microorganism associated with inflammatory changes increased the pathological score by one.

^c^Mixed infection: multiple pathogens are detected, and it is not possible to determine which one represents the etiological cause of death.

### Determination of the Cause of Death

Once all the analysis of the MIA samples had been completed, a panel composed of a pathologist, a microbiologist, and a clinician with expertise in infectious diseases and epidemiology evaluated all the data of the MIA and assigned the MIA diagnosis, i.e., the disease or condition putatively leading to death. No clinical information was used for the MIA diagnosis assignment. After a washout period (minimum 3 mo, range 3–6 mo), the same panel evaluated the data from the CDA and the clinical records, and assigned the final diagnosis of cause of death (CDA diagnosis). All morbid conditions directly leading to death, any underlying conditions (if present), as well as any other significant conditions possibly contributing to death were codified following ICD-10 (International Classification of Diseases and Related Health Problems 10th Revision) [15]. This codification process was conducted independently for the MIA and CDA diagnoses. To assess the reproducibility of the coding guidelines and the ICD-10 codification, a random sample of 15 CDAs were blindly coded by a second investigator who was not involved in the initial assignment of codes.

The causes of death were classified into four major groups of diseases: infectious diseases, malignant tumors, other diseases (including non-infectious cardiovascular, gastrointestinal, kidney, and lung diseases), and non-conclusive. When more than one severe pathological and/or microbiological diagnosis was identified, the disease most likely causing death was considered the CDA diagnosis. In all cases, the direct cause of death, and not the underlying disease, was considered as the main cause of death (e.g., miliary tuberculosis in a patient with HIV infection or myocardial infarction in a patient with severe atherosclerosis). The same coding system and criteria were applied to the MIA diagnoses.

Using a combination of the strength of the evidence of the histological and the microbiological findings, a category was assigned to the certainty of the cause of death attribution of the MIA diagnosis and the CDA diagnosis. These categories included no diagnosis and diagnosis of low, moderate, high, and very high certainty (Table 2). In the CDA evaluation, the clinical data were used to provide guidance and/or evidence on cause of death in cases with no diagnosis or with pathological/microbiological diagnoses of low or moderate certainty.

**Table 2:** Level of certainty of the diagnosis of cause of death obtained by combination of the strength of the evidence of the pathological and microbiological findings.

Pathology	Microbiology								
	0	1		2		3		4	
		N	Y	N	Y	N	Y	N	Y
**0** *	No diagnosis*	No diagnosis*		No diagnosis*		Low*		Moderate*	
**1**	Low	Low	Low	Low	Low	Moderate	Moderate	Moderate	Moderate
**2**	Low	Low	Low	Low	Moderate	Moderate	Moderate	Moderate	High
**3**	Moderate	Moderate	Moderate	Moderate	High	High	High	High	Very High
**4**	High	High	High	High	High	High	Very High	Very High	Very High

N: the microorganisms identified are rarely associated with the histological lesions observed; Y: the microorganisms identified are in concordance with the histological lesions observed.

*When the level of evidence for the pathology findings is zero, N and Y are not applicable.

**Fig 1:**
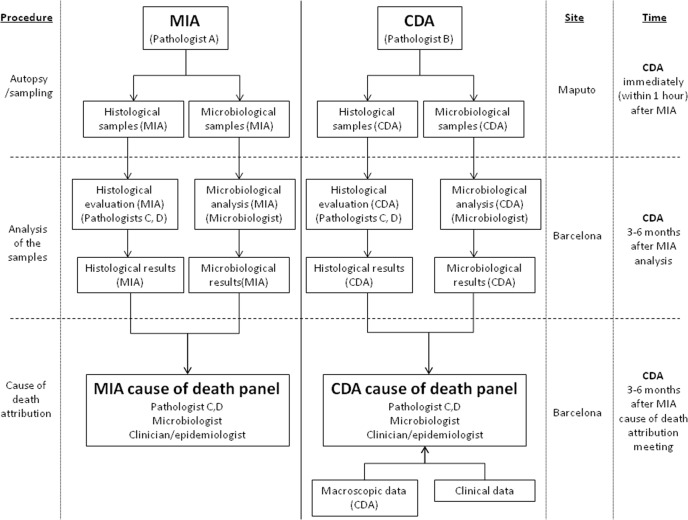
Overall study plan showing the procedures performed in the study, the investigators involved, and site and timing of each procedure. CDA, complete diagnostic autopsy; MIA, minimally invasive autopsy.

There were no differences between the planned and the final analysis of the samples performed, with the exception of the scales of the strength of the evidence and the levels of diagnostic certainty, which were developed during the process of sample analysis. Fig 1 illustrates the overall process and which investigators were involved at each stage.

### Statistical Methods

Proportions were compared by Fisher’s exact test, and odds ratios (ORs) were calculated as a measure of effect size whenever needed. The diagnostic efficacy of the MIA to identify the final CDA diagnosis was evaluated as sensitivity, specificity, and positive and negative predictive values. The association between the level of certainty of the MIA diagnosis and the concordance with the CDA diagnosis was evaluated by the Kendall tau-b rank correlation.

The concordance between the MIA and the CDA diagnosis was established by comparing ICD-10 codes, which classify diagnoses into nested classes of different hierarchical levels. In ICD-10, codes are organized in chapters, blocks, and three-character categories [15,16]. Thus, a concordance was classified as complete when the ICD-10 codes were identical in chapter, block, and three-character category [16]. Concordance was classified as partial when the codes were within the same chapter, but there was a discrepancy either in the block or the three-character category. Finally, when the MIA and the CDA diagnoses were in different chapters, the diagnoses were classified as discrepant.

The concordance between the MIA and the CDA diagnosis in terms of major groups of diseases was assessed by the kappa statistic (95% confidence interval from 1,000 bootstrap replications) and was interpreted as suggested by Landis and Koch [17,18]. Statistical analysis was performed using Stata version 14.1 (StataCorp).

The analytical plan was designed when the histological and microbiological results were available. The kappa statistic was included during the peer review process.

## Results

Coupled MIA and CDA procedures were performed in 112 adults (57 males and 55 females; median age 37 y, range 16–76). The interval between death and MIA and CDA ranged between 8 and 23 h. Seventy-three out of 112 patients (65.2%) tested positive for antibodies against HIV (all being HIV-1). The viral load was >50,000 copies/ml in 67 out of the 73 HIV-positive patients (91.8%).

### Minimally Invasive Autopsy Diagnosis and Complete Diagnostic Autopsy Diagnosis of Cause of Death

A MIA diagnosis of cause of death was obtained in 100 out of 112 (89.2%) cases. The level of certainty of the MIA diagnosis was considered low in 13/100 cases, moderate in 15/100 cases, and high or very high in 72/100 cases. A CDA diagnosis of cause of death was obtained in all cases. The certainty of the CDA diagnosis was low in 3/112 cases, moderate in 7/112 cases, and high or very high in 102/112 cases. In two cases, the pathological/microbiological analyses led to no diagnosis. In both cases, the CDA diagnosis was acute gastroenteritis with severe hydro-electrolytic disorder based on clinical information. Infectious diseases accounted for 71.4% (80/112) of all deaths. Patients with HIV infection died more frequently of infectious diseases than HIV-negative patients (60/73, 82.1%, versus 20/39, 52.3%, OR = 4.38 [95% CI: 1.69, 11.47], *p* = 0.001, Fisher’s exact test). In contrast, other diseases were less frequent in HIV-positive than in HIV-negative patients (4/73, 5.5%, versus 12/39, 30.8%, OR = 0.13 [95% CI: 0.03, 0.49], *p* < 0.001, Fisher’s exact test).

**Fig 2:**
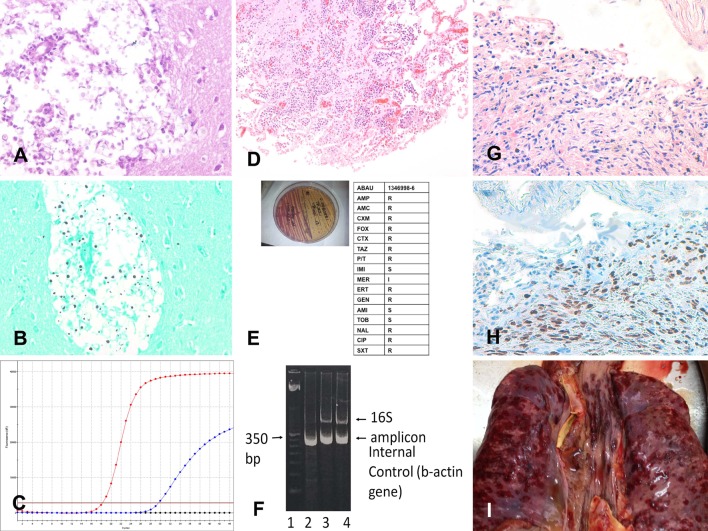
Three representative cases of cause of death identification by minimally invasive autopsy. (A–C) Cryptococcal encephalitis: (A) *Cryptococcus* spp. infecting the central nervous system (hematoxylin and eosin, 200×); (B) *Cryptococcus* spp. (methenamine silver stain, 200×); (C) real-time PCR positive for *Cryptococcus* spp. in the cerebrospinal fluid. (D–F) *Acinetobacter baumannii* pneumonia: (D) *A*. *baumannii* pneumonia infecting the lung (hematoxylin and eosin, 100×); (E) *A*. *baumannii* isolated (left side of the plate) and subjected to antibiotic susceptibility testing; (F) *A*. *baumannii* 16S RNA PCR amplification from tissue samples. (G–I) Disseminated Kaposi sarcoma: (G) Kaposi sarcoma involving the lung (hematoxylin and eosin, 100×); (H) Kaposi sarcoma involving the lung positive for human herpesvirus 8 (herpesvirus 8 antibody, 100×); (I) disseminated Kaposi sarcoma lesions in the lung (red areas on the pleural surface of the two lungs and the trachea; macroscopic image from the complete diagnostic autopsy).

Fig 2 shows three representative example cases of causes of death identified with the MIA.

### Concordance between the Minimally Invasive Autopsy Diagnosis and the Complete Diagnostic Autopsy Diagnosis

The assignment of ICD-10 codes was identical in the 15 CDAs selected for validation. Table 3 shows the CDA diagnoses of cause of death and the concordance of the MIA diagnoses with the CDA diagnoses. The MIA and CDA diagnoses were concordant in 85/112 (75.9%) cases. Concordance was complete in 77/112 (68.8%) and partial in 8/112 (7.1%) of the cases. Concordance between the MIA and CDA diagnoses was higher for infectious diseases (63/80; 78.8%) and tumors (13/16; 81.3%) than for other diseases (9/16; 56.2%). A discrepant diagnosis was observed in 27/112 (24.1%) of cases. Other diseases, including non-infectious cardiovascular, gastrointestinal, kidney, and lung diseases, were the most frequently missed conditions in the MIA (7/16; 43.7%). In the group of infectious diseases, gastrointestinal infections (2/2; 100%) and pulmonary infections (7/24; 29.2%) were the most frequently missed conditions. In the group of malignant tumors, carcinoma of the uterine cervix was the most frequently missed neoplasm (2/3; 66.6%).

**Table 3:** Causes of death determined by the complete diagnostic autopsy and concordance of the minimally invasive autopsy diagnosis with the complete diagnostic autopsy diagnosis.

Cause of Death in the Complete Diagnostic Autopsy	*N*	Minimally Invasive Autopsy Diagnosis Concordance			
		Complete		Partial	
		*N*	Percent	*N*	Percent
**Infectious diseases**	**80**	**61**	**76.3**	**2**	**2.5**
**Disseminated infections**	**41**	**35**	**85.4**	**0**	**0**
*Mycobacterium tuberculosis*	16	15	93.8	0	0
*Cryptococcus* spp.	6	6	100	0	0
Mixed*	3	3	100	0	0
*Toxoplasma gondii*	5	5	100	0	0
Enterobacteriaceae**	6	2	33.3	0	0
No etiology identified	2	1	50.0	0	0
Other***	3	3	100	0	0
**Pulmonary infections**	**24**	**15**	**62.5**	2	**8.3**
Enterobacteriaceae^†^	7	5	71.4	0	0
*Mycobacterium tuberculosis*	5	3	60.0	0	0
*Pneumocystis jirovecii*	2	2	100	0	0
Non-fermentative Gram-negative bacteria^††^	2	1	50.0	0	0
Mixed^†††^	2	1	50.0	1	50.0
No etiology identified	2	2	100	0	0
Other^††††^	4	1	25.0	1	25.0
**Central nervous system infections**	**13**	**11**	**84.6**	**0**	**0**
*Streptococcus pneumoniae*	3	3	100	0	0
*Mycobacterium tuberculosis*	2	1	50.0	0	0
*Toxoplasma gondii*	2	2	100	0	0
Other^₹^	6	5	83.3	0	0
**Gastrointestinal infections**	**2**	**0**	**0**	**0**	**0**
No etiology identified	2	0	0	0	0
**Malignant tumors**	**16**	**13**	**81.3**	**0**	**0**
Hepatocellular carcinoma	5	5	100	0	0
Carcinoma of the uterine cervix	3	1	33.3	0	0
Malignant lymphoma	3	3	100	0	0
Kaposi sarcoma	2	2	100	0	0
Other tumors	3	2	66.7	0	0
**Other diseases**	**16**	**3**	**18.8**	**6**	**37.5**
Complications of cardiovascular diseases^‡^	11	1	9.1	6	54.5
Lung diseases^‡‡^	2	2	100	0	0
Gastrointestinal and kidney diseases^‡‡‡^	3	0	0	0	0

*One case *Escherichia coli* + *Lactobacillus* sp., one case *Acinetobacter* sp. + *Enterobacter* sp., and one case *Prevotella* spp. + *Streptococcus pneumoniae* + human herpesvirus 1 + cytomegalovirus + *Toxoplasma gondii*.

**Three cases *E*. *coli*, one case *Klebsiella*. *pneumoniae*, one case *Salmonella typhi*, and one case *Enterobacter* spp.

***One case *Candida glabrata*, one case human herpesvirus 1, and one case *Streptococcus dysgalactiae*.

^†^Four cases *K*. *pneumoniae*, two cases *E*. *coli*, and one case *Enterobacter* spp.

^††^One case *Pseudomona aeruginosa* and one case *Acinetobacter baumannii*.

^†††^One case adenovirus + *Cryptococcus neoformans* and one case cytomegalovirus + human herpesvirus-1.

^††††^One case *Legionella*. *pneumophila*, one case *Mycoplasma* spp., one case *T*. *gondii*, and one case adenovirus.

^₹^One case *Cryptococcus* spp., one case mucormycosis, one case cytomegalovirus, one case human herpesvirus 1, one case rabies, and one case of *Prevotella* spp.

^‡^Complications of cardiovascular diseases include five cases of cerebral infarction/hemorrhage, two cases of diabetic ketoacidosis, two cases of myocardial infarction, one case of dilated myocardiopathy, and one case of hypertensive renal disease with renal failure.

^‡‡^One case of pulmonary fibrosis with pulmonary hypertension and one case of pneumoconiosis.

^‡‡‡^One case of acute thrombotic microangiopathy with renal necrosis, one case of alcoholic cirrhosis with upper gastrointestinal hemorrhage, and one case of gastric ulcer with upper gastrointestinal hemorrhage.

Table 4 shows the sensitivity, specificity, and the positive and negative predictive values of the MIA diagnosis for the major diagnostic categories, as well as the percentage of false-positive and false-negative diagnoses and of cases correctly classified by the MIA. The level of certainty of the MIA diagnosis was clearly associated with the concordance with the CDA diagnosis (Kendall tau-b rank correlation coefficient = 0.5257, *p* < 0.001).

**Table 4:** Sensitivity, specificity, and positive and negative predictive values and percentage of false-positive and false-negative diagnoses and cases correctly classified by the minimally invasive autopsy.

Cause of Death	*n*	Sensitivity	Specificity	Positive Predictive Value	Negative Predictive Value	Percent False Positive	Percent False Negative	Percent Correctly Classified
Disseminated infections	41	98 (87, 100)	99 (92, 100)	98 (87, 100)	99 (92, 100)	1 (0, 8)	2 (0, 13)	98 (94, 100)
Pulmonary infections	24	79 (58, 93)	99 (94, 100)	95 (75, 100)	95 (88, 98)	1 (0, 6)	21 (7, 42)	95 (90, 99)
Central nervous system infections	13	92 (64, 100)	100 (96, 100)	100 (74, 100)	99 (95, 100)	0 (0, 4)	8 (0, 36)	99 (95, 100)
Gastrointestinal infections	2	0 (0, 84)	100 (97, 100)		98 (94, 100)	0 (0, 3)	100 (16, 100)	98 (94, 100)
Malignant tumors	16	81 (54, 96)	100 (96, 100)	100 (75, 100)	97 (91, 99)	0 (0, 4)	19 (4, 46)	97 (94, 100)
Other diseases	16	69 (41, 89)	98 (93, 100)	85 (55, 98)	95 (89, 98)	2 (0, 7)	31 (11, 59)	94 (89, 98)

Figures are given as percentage (95% confidence interval).

An etiological agent was identified in the CDA in 74/80 (92.5%) of the patients dying from an infectious cause. The microorganisms identified are shown in Table 3. The same microorganism was identified in the MIA in 62/74 (83.8%) patients. In four patients, the MIA diagnosis was based only on the results of the microbiological analyses. All but one of the five hepatocellular carcinomas were positive for hepatitis B virus. The fifth was negative for hepatitis B virus and hepatitis C virus. Human papillomavirus type 16 was identified in two carcinomas of the uterine cervix, and human papillomavirus type 35 in one case. The CDA diagnosis and the MIA diagnosis of each case are shown in S1 Table.

Table 5 shows the correlation between the MIA diagnosis and the CDA diagnosis of all cases, grouped according to the major disease categories. The observed agreement was 86.6%. As the expected agreement by chance (but with probabilities equal to the overall proportions) is 50%, the observed agreement was 73.2% (kappa = 0.732 [95% CI: 0.615, 0.838]; substantial agreement according to the Landis and Koch classification).

**Table 5:** Correlation between the minimally invasive autopsy diagnosis and the complete diagnostic autopsy diagnosis of all cases, grouped according to the major disease categories.

Minimally Invasive Autopsy	Complete Diagnostic Autopsy				Total
	Infection	Tumor	Other	Non-conclusive	
**Infection**	72	0	1	0	73
**Tumor**	0	13	0	0	13
**Other**	2	0	12	0	14
**Non-conclusive**	6	3	3	0	12
**Total**	80	16	16	0	112

### Underlying Conditions and Associated Lesions/Concomitant Infections

HIV infection was identified as the underlying cause of death in 67 cases (59.8%). HIV infection was detected in six additional patients dying of diseases not related to HIV, and, consequently, HIV infection was considered an associated condition in these cases. All the HIV infections were captured in the MIA. An underlying condition other than HIV infection was identified in 22 cases (see S1 Table). Associated lesions not related to the sequence of events directly leading to death were identified in 81/112 (72.3%) of the cases. Fifty-seven of these (70.4%) were identified by the MIA. No active malaria was identified, and PCR analysis for *P*. *falciparum* was negative in all cases, but four patients had histological evidence of previous malaria (hemozoin in liver macrophages). Three patients had liver schistosomiasis.

## Discussion

This study shows that an easy, rapid, and non-disfiguring standardized minimally invasive sampling procedure designed for postmortem studies in Mozambique may provide a correct diagnosis in the majority of cases. This validation study shows a high degree of concordance (75.9%; kappa = 0.732) between the MIA and CDA diagnoses in a series of adults who died at a quaternary hospital in Mozambique. These findings are important since they open a new pathway for cause of death investigation in places where postmortem methods have not traditionally been used. This method may improve the current capacities to conduct cause of death surveillance in large parts of the world where mortality remains high but knowledge of what people die of is currently based on assumptions.

In this study, the concordance of the MIA diagnosis with the CDA diagnosis was almost 80% for infectious diseases. These results are similar to a few recent reports using similar approaches in HIV-positive/AIDS patients [19,20]. The leading cause of infectious deaths in the current study was *M*. *tuberculosis*, a finding consistent with previous autopsy studies from sub-Saharan Africa [19–22]. Interestingly, the MIA protocol consistently provided good quality tissues for adequate microbiological analyses, allowing confirmation of the pathological results and etiological characterization of the microorganisms causing death in over 86% of the cases. Furthermore, the microbiology analyses increased the level of confidence in the diagnosis when the strength of the pathological findings was low. Although the number of cases is limited, the two gastrointestinal infections identified as the cause of death by the CDAs were not identified by the MIAs. Sampling of stool and/or bowel mucosa could be included in the protocol in order to improve these results [12,13]. However, further studies are necessary to evaluate the usefulness of stool analysis in postmortem studies. Additionally, the MIA was relatively less efficient for the diagnosis of pulmonary infections, which were missed in 29.2% of the cases, reflecting the relatively lower efficiency of the needle sampling for the lung [12,13].

The MIA diagnosis was also highly accurate for malignant tumors, especially for hepatocellular carcinoma and Kaposi sarcoma, two of the most prevalent tumors in Mozambique according to a recent report [23]. Identification of other non-infectious diseases (including cardiovascular, lung, gastrointestinal, and kidney diseases) was less accurate using the MIA procedure. Diagnosis of such entities, however, remains challenging even with the CDA due to the variety of lesions and organs involved. These cases often require a combination of all the macroscopic data available and frequently the clinical information, which was not used in our study, to complete the non-conclusive pathological results [24–26].

Innovative strategies have been developed to overcome the low acceptability and feasibility of the CDA. Ideally, these alternative postmortem examination methods should be capable of providing results comparable to conventional CDA. Imaging-based methods using magnetic resonance imaging, computerized scans, or ultrasounds, frequently combined with needle biopsies, have been all shown to provide accurate results [27,28]. However, costs, reliance on sophisticated equipment, and mandatory involvement of highly skilled personnel are critical limitations of these strategies that hamper their introduction in low-income settings [29]. In recent years, different approaches based only on minimally invasive tissue sampling [30–32] have been proposed as potential substitutes for CDA. This MIA is expected to be more acceptable to the relatives of the deceased person than the CDA, especially in rural areas, where most deaths still occur in low-income countries [33]. In addition, the MIA procedure likely involves less risk than the CDA for health personnel, something of critical importance when faced with highly contagious infectious diseases. Finally, the MIA procedure could easily be performed by trained technicians, which might enable widespread use of this method in low-income countries in the absence of a sufficient pathologist workforce.

A possible limitation of the MIA is that its diagnostic accuracy may be influenced by the dissemination of the disease: the performance of the procedure may be significantly reduced in focal lesions and in limited infections in immunocompetent hosts. In this study, more than half of the patients were HIV-infected adults with disseminated infections, which might have increased the diagnostic yield of the MIA. Additionally, the study population included only hospitalized patients, whose causes of death may not reflect accurately the predominant causes of death occurring in the community. A limitation for its potential use in rural areas is that recruitment of cases into the study was restricted to the first 24 h after death. Given that a significant proportion of deaths in low-income countries occur at home, it is plausible that access to those bodies could possibly be delayed beyond the first 24 h, which may affect the performance of the MIA. Both the MIA and the CDA include a degree of expert subjective interpretation. In the present study, both evaluations were made by the same group of experts in order to focus on the differences between the two methods, thus minimizing the differences in expertise between observers. As a consequence, the study design can support internal consistency, but not necessarily external generalizability, particularly given the subjective nature of the histological interpretation. Finally, limited experience in pathology (histochemistry, immunohistochemistry) and microbiology (molecular diagnostics) laboratories and restricted resources may be a limitation for both CDA and MIA implementation in low-income settings.

In conclusion, the results of this study confirm that the MIA procedure is a valid tool comparable to the gold standard, CDA, for cause of death determination in adult deaths in Mozambique. This method could have an important role in determining the cause of death in middle- and low-income countries where accurate assessment of the causes of mortality is virtually nonexistent and where infectious diseases are extremely frequent. The diagnostic accuracy of the MIA for cause of death determination needs to be assessed also in specific vulnerable groups, including in pediatric and maternal deaths. The use of this tool could improve health planning and priority setting and, ultimately, improve the duration of healthy lives for the most vulnerable populations in the world.

## Supporting Information

S1 Table Diagnoses obtained by complete diagnostic autopsy (final diagnosis) and minimally invasive autopsy (putative diagnosis) for each case and degree of concordance between the putative and the final diagnosis. For each method, the cause of death (final and putative diagnoses), the underlying conditions and other significant conditions, and the ICD-10 codes are shown. The level of certainty of the putative and final diagnoses as well as the strength of the evidence of the pathological and microbiological findings are also shown.
(DOCX)

S1 Text STROBE checklist for the observational study developed in Mozambique to validate the minimally invasive autopsy for cause of death determination in adults. (DOCX)

S2 Text Prospective analysis plan for pathological and microbiological procedures used during the study. (DOCX)

## Abbreviations

CDA: complete diagnostic autopsy
CNS: central nervous system
CSF: cerebrospinal fluid
MIA: minimally invasive autopsy
OR: odds ratio
