# Supplementary material for: Validity of a Minimally Invasive Autopsy for Cause of Death Determination in Adults in Mozambique: An Observational Study
Source: PLoS Med. 2016 Nov 22;13(11):e1002171. doi: 10.1371/journal.pmed.1002171 (PMC5119723; doi:10.1371/journal.pmed.1002171)
Supplement: S1 Table — For each method, the cause of death (final and putative diagnoses), the underlying conditions and other significant conditions, and the ICD-10 codes are shown. The level of certainty of the putative and final diagnoses as well as the strength of the evidence of the pathological and microbiological findings are also shown. (DOCX) [file pmed.1002171.s001.docx]

|  | **Complete diagnostic autopsy (CDA) results** | | | |  |  |  |  | **Minimally invasive autopsy (MIA) results** | | | | | |  |  |
| --- | --- | --- | --- | --- | --- | --- | --- | --- | --- | --- | --- | --- | --- | --- | --- | --- |
| **Case Num.** | **Final Diagnosis** | **ICD-10** | **Underlying conditions** | **ICD-10** | **Other significant conditions** | **ICD-10** | **Level of certainty (strength of the evidence pathology + microbiology) ^(a)^** |  | **Putative diagnosis (b)** | **ICD-10** | **Underlying condition** | **ICD-10** | **Other significant condition** | **ICD-10** | **Level of certainty (strength of the evidence pathology + microbiology) ^(b)^** | **Concordance** |
| **Infectious diseases** | | | |  |  |  |  |  |  |  |  |  |  |  |  |  |
| **Disseminated infections** | |  |  |  |  |  |  |  |  |  |  |  |  |  |  |  |
|  | ***Mycobacterium tuberculosis*** | |  |  |  |  |  |  |  |  |  |  |  |  |  |  |
| 10 | Milliary tuberculosis | A19 | HIV | B20.0 |  |  | Very high (4+4) |  | Milliary tuberculosis | A19 | HIV | B20.0 |  |  | Very high (4+4) | Complete |
| 12 | Milliary tuberculosis | A19 | HIV | B20.0 |  |  | Very high (4+4) |  | Milliary tuberculosis | A19 | HIV | B20.0 |  |  | Very high (4+4) | Complete |
| 24 | Milliary tuberculosis | A19 | HIV | B20.0 |  |  | Very high (4+4) |  | Milliary tuberculosis | A19 | HIV | B20.0 |  |  | Very high (4+4) | Complete |
| 50 | Milliary tuberculosis | A19 | HIV | B20.0 |  |  | Very high (4+4) |  | Milliary tuberculosis | A19 | HIV | B20.0 |  |  | Very high (4+4) | Complete |
| 53 | Milliary tuberculosis | A19 | HIV | B20.0 |  |  | Very high (4+4) |  | Milliary tuberculosis | A19 | HIV | B20.0 |  |  | Very high (4+4) | Complete |
| 57 | Milliary tuberculosis | A19 | HIV | B20.0 | Cirrhosis | K74 | Very high (4+4) |  | Cirrhosis | K74 |  |  | HIV | B24 | High (4+0) | Discrepant |
| 63 | Milliary tuberculosis | A19 | HIV | B20.0 |  |  | Very high (4+4) |  | Milliary tuberculosis | A19 | HIV | B20.0 |  |  | Very high (4+4) | Complete |
| 64 | Milliary tuberculosis | A19 | HIV | B20.0 |  |  | Very high (4+4) |  | Milliary tuberculosis | A19 | HIV | B20.0 |  |  | Very high (4+4) | Complete |
| 67 | Milliary tuberculosis | A19 | HIV | B20.0 |  |  | Very high (4+4) |  | Milliary tuberculosis | A19 | HIV | B20.0 |  |  | Very high (4+4) | Complete |
| 68 | Milliary tuberculosis | A19 | HIV | B20.0 |  |  | Very high (4+4) |  | Milliary tuberculosis | A19 | HIV | B20.0 |  |  | Very high (4+4) | Complete |
| 69 | Milliary tuberculosis | A19 | HIV | B20.0 |  |  | Very high (4+4) |  | Milliary tuberculosis | A19 | HIV | B20.0 |  |  | Very high (4+4) | Complete |
| 73 | Milliary tuberculosis | A19 | HIV | B20.0 |  |  | Very high (4+4) |  | Milliary tuberculosis | A19 | HIV | B20.0 |  |  | Very high (4+4) | Complete |
| 74 | Milliary tuberculosis | A19 | HIV | B20.0 |  |  | Very high (4+4) |  | Milliary tuberculosis | A19 | HIV | B20.0 |  |  | Very high (4+4) | Complete |
| 75 | Milliary tuberculosis | A19 | HIV | B20.0 |  |  | Very high (4+4) |  | Milliary tuberculosis | A19 | HIV | B20.0 |  |  | Very high (4+4) | Complete |
| 77 | Milliary tuberculosis | A19 | HIV | B20.0 |  |  | Very high (4+4) |  | Milliary tuberculosis | A19 | HIV | B20.0 |  |  | Very high (4+4) | Complete |
| 78 | Milliary tuberculosis | A19 | HIV | B20.1 |  |  | Very high (4+4) |  | Milliary tuberculosis | A19 | HIV | B20.0 |  |  | Very high (4+4) | Complete |
|  | ***Cryptococcus sp.*** | |  |  |  |  |  |  |  |  |  |  |  |  |  |  |
| 13 | Sepsis (Cryptococcus sp.) | B45.7 | HIV | B20.8 |  |  | Very high (4+4) |  | Sepsis (*Cryptococcus sp.*) | B45.7 | HIV | B20.8 |  |  | Very high (4+4) | Complete |
| 22 | Sepsis (Cryptococcus sp.) | B45.7 | HIV | B20.8 |  |  | Very high (4+4) |  | Sepsis (*Cryptococcus sp.*) | B45.7 | HIV | B20.8 |  |  | Very high (4+4) | Complete |
| 25 | Sepsis (Cryptococcus sp.) | B45.7 | HIV | B20.8 |  |  | Very high (4+4) |  | Sepsis (*Cryptococcus sp.*) | B45.7 | HIV | B20.8 |  |  | Very high (4+4) | Complete |
| 36 | Sepsis (Cryptococcus sp.) | B45.7 | HIV | B20.8 | Chronic viral hepatitis (HBV) | B18 | Very high (4+4) |  | Sepsis (*Cryptococcus sp.*) | B45.7 | HIV | B20.8 | Chronic viral hepatitis (HBV) | B18 | Very high (4+3) | Complete |
| 42 | Sepsis (Cryptococcus sp.) | B45.7 | HIV | B20.8 |  |  | Very high (4+4) |  | Sepsis (*Cryptococcus sp.*) | B45.7 | HIV | B20.8 |  |  | Very high (4+4) | Complete |
| 44 | Sepsis (Cryptococcus sp.) | B45.7 | HIV | B20.8 |  |  | Very high (4+4) |  | Sepsis (*Cryptococcus sp.*) | B45.7 | HIV | B20.8 |  |  | Very high (4+4) | Complete |
|  | **Mixed** | |  |  |  |  |  |  |  |  |  |  |  |  |  |  |
| 17 | Sepsis (mixed HSV-1+ *Prevotella spp*. + *T. gondii* + cytomegalovirus + *S. pneumoniae*) | A41 | HIV | B20.1 |  |  | High (4+2) |  | Sepsis (mixed HVS-1 + *Prevotella spp.* + *T. gondii* + Cytomegalovirus + *S. pneumoniae*) | A41 | HIV | B20.1 |  |  | High (4+2) | Complete |
| 34 | Sepsis of biliary origin (*E. coli*, *Lactobacillus sp*) | A41 | Toxic liver disease with cholestasis | K71.0 |  |  | High (4+2) |  | Sepsis of biliary origin (*E. coli*, *Lactobacillus sp*) | A41 | Toxic liver disease with cholestasis | K71.0 |  |  | Moderate (2+2) | Complete |
| 38 | Sepsis of abdominal origin (*Acinetobacter sp*, *Enterobacter sp*) | A41 | Diabetes Mellitus | E14 |  |  | High (4+2) |  | Sepsis (*Acinetobacter sp*, *Enterobacter sp*) | A41 | Peritonitis | K65 |  |  | High (3+2) | Complete |
|  | ***Toxoplasma gondii*** | |  |  |  |  |  |  |  |  |  |  |  |  |  |  |
| 3 | Disseminated toxoplasmosis | B58.9 | HIV | B20.8 |  |  | Very high (4+4) |  | Disseminated toxoplasmosis | B58.9 | HIV | B20.8 |  |  | Very high (3+4) | Complete |
| 20 | Disseminated toxoplasmosis | B58.9 | HIV | B20.8 |  |  | Very high (4+4) |  | Cerebral toxoplasmosis | B58.2 | HIV | B20.8 |  |  | Very high (3+4) | Complete |
| 45 | Disseminated toxoplasmosis | B58.9 | HIV | B20.8 | Pneumonia (*Pneumocystis jirovecii*) | B59 | Very high (4+4) |  | Disseminated toxoplasmosis | B58.9 | HIV | B20.8 | Pneumonia (*Pneumocystis jirovecii*) | B59 | Very high (4+4) | Complete |
| 71 | Disseminated toxoplasmosis | B58.9 | HIV | B20.8 |  |  | Very high (4+4) |  | Disseminated toxoplasmosis | B58.9 | HIV | B20.8 |  |  | Very high (4+4) | Complete |
| 72 | Disseminated toxoplasmosis | B58.9 | HIV | B20.8 |  |  | Very high (4+4) |  | Cerebral toxoplasmosis | B58.2 | HIV | B20.8 |  |  | Very high (3+4) | Complete |
|  | ***Enterobacteriaceae*** | |  |  |  |  |  |  |  |  |  |  |  |  |  |  |
| 21 | Sepsis of urinary origin (*E. coli*) | A41.5 | HIV | B20.1 | Diabetes Mellitus 2 | E11 | High (4+2) |  | Sepsis of urinary origin (*E. coli*) | A41.5 | HIV | B20.1 |  |  | Very high (4+3) | Complete |
| 33 | Sepsis of urinary origin (*E. coli*) | A41.5 | HIV | B20.1 |  |  | Very high (4+4) |  | Pneumonia (*E.coli*) | J15.5 | HIV | B20.1 |  |  | Very high (4+4) | Discrepant |
| 40 | Sepsis (*S. typhi*) | A02.1 | HIV | B20.1 |  |  | Very high (4+4) |  | Sepsis (*S. typhi*) | A02.1 | HIV | B20.1 |  |  | Moderate (0+4) | Complete |
| 43 | Sepsis (*E. coli*) | A41.5 | HIV | B20.1 | Pulmonary tuberculosis | A15 | High (4+1) |  | Pneumonia (*S. aureus*, *E. coli*, *K. pneumoniae*) | J16 | HIV | B20.1 |  |  | High (4+2) | Discrepant |
| 52 | Sepsis (*K. pneumoniae*) | A45.1 |  |  | Cachexia | R64 | Very high (4+3) |  | Meningoencephalitis | G04 |  |  | Cachexia | R64 | Low (1+0) | Discrepant |
| 61 | Sepsis (*Enterobacter spp.*) | A41.5 |  |  |  |  | High (4+2) |  | Pneumonia (*Enterobacter spp.*) | J15.6 |  |  |  |  | High (4+2) | Discrepant |
|  | **No etiology identified** | |  |  |  |  |  |  |  |  |  |  |  |  |  |  |
| 18 | Sepsis | A41 | HIV | B20.1 | Cirrhosis | K74 | High (4+0) |  | Meningoencephalitis (mixed HIV, *K. pneumoniae*) | G00 | HIV | B20.1 | Cirrhosis | K74 | High (4+2) | Discrepant |
| 54 | Sepsis | A41 |  |  | Pulmonary tuberculosis | A15 | High (4+0) |  | Sepsis | A41 |  |  |  |  | High (4+0) | Complete |
|  | **Other** | |  |  |  |  |  |  |  |  |  |  |  |  |  |  |
| 14 | Sepsis (*S. dysgalactiae*) | A40.1 | HIV | B24 |  |  | Very high (4+4) |  | Sepsis (*S. dysgalactiae*) | A40.1 | HIV | B24 |  |  | High (2+4) | Complete |
| 29 | Disseminated infection (HHV-1) | B00.7 | HIV | B20.3 |  |  | Very high (4+4) |  | Pneumonia (HHV-1) | B00.7 | HIV | B20.3 |  |  | High (4+2) | Complete |
| 35 | Sepsis of appendicular origin (*C. glabrata*) | B37.7 | Appendicitis (acute) with peritonitis following perforation | K35.2 | HIV | B20.8 | Very high (4+3) |  | Pneumonia (*C. glabrata*) | B37.1 | HIV | B20.8 |  |  | High (4+1) | Complete |
| **Pulmonary infections** | |  |  |  |  |  |  |  |  |  |  |  |  |  |  |  |
|  | ***Enterobacteriaceae*** | |  |  |  |  |  |  |  |  |  |  |  |  |  |  |
| 16 | Pneumonia (*K. pneumoniae*) | J15.0 | HIV | B20.1 |  |  | High (4+2) |  | Pneumonia (*K. pneumoniae*) | J15.0 | HIV | B20.1 |  |  | Very high (4+3) | Complete |
| 23 | Pneumonia (*E. coli*) | J15.5 | HIV | B20.1 |  |  | High (4+3) |  | Non-conclusive | R99 | HIV | B20.1 |  |  | No diagnosis (0+2) | Discrepant |
| 26 | Pneumonia (*K. pneumoniae*) | J15.0 | HIV | B20.1 |  |  | High (4+2) |  | Pneumonia (*K. pneumoniae*) | J15.0 | HIV | B20.1 |  |  | High (3+3) | Complete |
| 49 | Pleuropericarditis (*K. pneumoniae*) | I30.9 |  |  |  |  | High (4+2) |  | Pleuritis (*K. pneumoniae*) | I30.9 |  |  |  |  | Moderate (1+3) | Complete |
| 58 | Pneumonia (*K. pneumoniae*) | J15.0 | HIV | B20.1 |  |  | High (4+2) |  | Pneumonia (*K. pneumoniae* and rhinovirus) | J15.0 | HIV | B20.1 |  |  | Very high (4+4) | Complete |
| 59 | Pneumonia (*E. coli*) | J15.5 | HIV | B20.1 | Papillary renal cell carcinoma | C64 | High (4+2) |  | Suggestive of cardiovascular disease | I51.6 |  |  | HIV | B24 | Low (1+0) | Discrepant |
| 65 | Pneumonia (*Enterobacter spp.*) | J15.6 |  |  | Hypertensivecardiopathy and nephropathy |  | Moderate (3+2) |  | Pneumonia (*Enterobacter spp.*) | J15.6 |  |  |  |  | Moderate (3+1) | Complete |
|  | ***Mycobacterium tuberculosis*** | |  |  |  |  |  |  |  |  |  |  |  |  |  |  |
| 30 | Pulmonary tuberculosis | A15 |  |  | Diabetes Mellitus 2 | E11 | Very high (4+4) |  | Pulmonary tuberculosis | A15 |  |  |  |  | Very high (4+4) | Complete |
| 37 | Tuberculosis with necrotizing pneumonia | A15 | HIV | B20.0 |  |  | Very high (4+4) |  | Non-conclusive | R99 | HIV | B24 |  |  | No diagnosis (0+0) | Discrepant |
| 39 | Tuberculosis with necrotizing pneumonia | A15 |  |  |  |  | Very high (4+4) |  | Non-conclusive | R99 |  |  |  |  | No diagnosis (0+0) | Discrepant |
| 62 | Pulmonary tuberculosis | A15 |  |  |  |  | Very high (4+4) |  | Pulmonary tuberculosis | A15 |  |  |  |  | Very high (4+4) | Complete |
| 76 | Pulmonary tuberculosis | A15 |  |  |  |  | Very high (4+4) |  | Pulmonary tuberculosis | A15 |  |  |  |  | Moderate (1+3) | Complete |
|  | ***Pneumocystis jirovecii*** | |  |  |  |  |  |  |  |  |  |  |  |  |  |  |
| 2 | Pneumonia (*Pneumocystis jirovecii*) | B59 | HIV | B20.6 | Cirrhosis | K74 | Very high (4+4) |  | Pneumonia (*Pneumocystis jirovecii*) | B59 | HIV | B20.6 | Cirrhosis | K74 | Very high (4+4) | Complete |
| 8 | Pneumonia (*Pneumocystis jirovecii*) | B59 | HIV | B20.6 |  |  | Very high (4+4) |  | Pneumonia (*Pneumocystis jirovecii*) | B59 | HIV | B20.6 |  |  | Very high (4+4) | Complete |
|  | **Mixed** | |  |  |  |  |  |  |  |  |  |  |  |  |  |  |
| 9 | Necrotizing pneumonia (Adenovirus) | J12.0 | HIV | B20.3 | Renal abscess | N15.1 | Very high (4+4) |  | Pneumonia | J18 | HIV | B20.3 |  |  | High (4+0) | Partial |
| 32 | Pneumonia (CMV and HHV-1) | B25.0 | HIV | B20.3 |  |  | High (4+3) |  | Meningoencephalitis (CMV and HHV-1) | B25.9 | HIV | B20.3 |  |  | Moderate (2+3) | Partial |
|  | **Non-fermentative gram-negative bacteria** | |  |  |  |  |  |  |  |  |  |  |  |  |  |  |
| 4 | Pneumonia (*Acinetobacter baumannii*) | J15 | HIV | B20.1 |  |  | Very high (4+4) |  | Pneumonia (*Acinetobacter baumannii*) | J15 | HIV | B20.1 |  |  | Very high (4+4) | Complete |
| 70 | Necrotizing bronchopneumonia (*Pseudomonas aeruginosa*) | J15.1 | HIV | B20.1 |  |  | Very high (4+3) |  | Sepsis (*Pseudomonas aeruginosa*) | A41.5 | HIV | B20.1 |  |  | Moderate (0+4) | Discrepant |
|  | **No etiology identified** | |  |  |  |  |  |  |  |  |  |  |  |  |  |  |
| 19 | Pneumonia | J18 | HIV | B20,1 | Chronic hepatitis | K75.9 | High (4+0) |  | Pneumonia | J18 | HIV | B20.1 |  |  | High (4+0) | Complete |
| 66 | Pneumonia | J18 | HIV | B20.1 |  |  | Moderate (3+0) |  | Pneumonia | J18 | HIV | B20.1 |  |  | High (4+0) | Complete |
|  | **Other** | |  |  |  |  |  |  |  |  |  |  |  |  |  |  |
| 46 | Pulmonary necrotizing toxoplasmosis | B58.3 |  |  | Hypertensivecardiopathy and nephropathy | I13 | Very high (4+4) |  | Pneumonia | J18 |  |  | Hypertensivecardiopathy and nephropathy | I13 | High (4+0) | Discrepant |
| 47 | Necrotizing pneumonia (*Legionella pneumophila*) | A48.1 | HIV | B20.1 |  |  | Very high (4+4) |  | Necrotizing pneumonia (*Legionella pneumophila*) | A48.1 | HIV | B20.1 |  |  | Very high (4+4) | Complete |
| 56 | Pneumonia (*Mycoplasma sp.*) | J15.7 |  |  |  |  | High (4+2) |  | Pneumonia | J18 |  |  |  |  | High (4+0) | Partial |
| 60 | Fibrinous hemorrhagic pericarditis (Adenovirus) | I30.1 |  |  |  |  | High (4+1) |  | Sepsis (*Enterobacter spp*, *K. pneumoniae*) | A41 |  |  |  |  | Moderate (3+2) | Discrepant |
| **Central nervous system infections** | | |  |  |  |  |  |  |  |  |  |  |  |  |  |  |
|  | ***Streptococcus pneumoniae*** | |  |  |  |  |  |  |  |  |  |  |  |  |  |  |
| 5 | Meningitis (*Streptococcus pneumoniae*) | G00.1 | HIV | B20.1 |  |  | Very high (4+4) |  | Meningitis (*Streptococcus pneumoniae*) | G00.1 | HIV | B20.1 |  |  | Very high (4+4) | Complete |
| 28 | Meningitis (*Streptococcus pneumoniae*) | G00.1 | HIV | B20.1 | Chronic hepatitis | K75.9 | Very high (4+4) |  | Meningitis (*Streptococcus pneumoniae*) | G00.1 | HIV | B20.1 | Chronic hepatitis | K75.9 | Very high (3+4) | Complete |
| 41 | Meningitis (*Streptococcus pneumoniae*) | G00.1 |  |  | Cirrhosis | K74 | Very high (4+4) |  | Meningitis (*Streptococcus pneumoniae*) | G00.1 |  |  | Cirrhosis | K74 | Moderate (1+4) | Complete |
|  | ***Mycobacterium tuberculosis*** | |  |  |  |  |  |  |  |  |  |  |  |  |  |  |
| 7 | Meningitis (*Mycobacterium tuberculosis*) | A17.0 | HIV | B20.0 |  |  | Very high (4+4) |  | Meningitis (*Mycobacterium tuberculosis*) | A17.0 | HIV | B20.0 |  |  | Very high (4+4) | Complete |
| 11 | Meningitis (*Mycobacterium tuberculosis*) | A17.0 |  |  |  |  | Very high (4+4) |  | Meningoencephalitis | G04 |  |  |  |  | Low (1+0) | Discrepant |
|  | ***Toxoplasma gondii*** | |  |  |  |  |  |  |  |  |  |  |  |  |  |  |
| 15 | Cerebral toxoplasmosis | B58.2 | HIV | B20.8 |  |  | Very high (4+4) |  | Cerebral toxoplasmosis | B58.2 | HIV | B20.8 |  |  | Moderate (0+4) | Complete |
| 55 | Cerebral Toxoplasmosis | B58.2 | HIV | B20.8 |  |  | Very high (4+4) |  | Cerebral toxoplasmosis | B58.2 | HIV | B20.8 |  |  | Moderate (0+4) | Complete |
|  | **Other** | |  |  |  |  |  |  |  |  |  |  |  |  |  |  |
| 1 | Rhizopus oryzae cerebral mucormycosis | B46.5 | Diabetes Mellitus 2 | E11 |  |  | Very high (4+4) |  | *Rhizopus oryzae* cerebral mucormycosis | B46.5 | Cutaneous abscess of scalp | L02.8 |  |  | Very high (4+4) | Complete |
| 6 | Meningoencephalitis (CMV) | B25.9 | HIV | B20.2 |  |  | High (4+3) |  | Meningoencephalitis (CMV) | B25.9 | HIV | B20.2 |  |  | High (4+3) | Complete |
| 27 | Meningoencephalitis (*Prevotella sp.*) | G00 |  |  |  |  | Very high (4+4) |  | Meningoencephalitis (*Prevotella sp.*) | G00 |  |  |  |  | Moderate (2+3) | Complete |
| 31 | Meningoencephalitis (HHV-1) | B00.3 |  |  |  |  | Very high (4+4) |  | Meningoencephalitis (HHV-1) | B00.3 |  |  |  |  | Very high (4+4) | Complete |
| 48 | Meningoencephalitis (*Cryptococcus sp.*) | B45.1 | HIV | B20.8 |  |  | Very high (4+4) |  | Meningoencephalitis (*Cryptococcus sp.*) | B45.1 | HIV | B20.8 |  |  | Very high (4+4) | Complete |
| 51 | Rabies | A82 |  |  |  |  | Moderate (0+4) |  | Non-conclusive | R99 |  |  |  |  | No diagnosis (0+0) | Discrepant |
| **Gastrointestinal infections** | | | | | | | |  |  |  |  |  |  |  |  |  |
| 111 | Gastroenteritis | A09 |  |  |  |  | low (2+0) |  | Non-conclusive | R99 |  |  |  |  | No diagnosis (0+0) | Discrepant |
| 112 | Gastroenteritis | A09 | HIV | B24 |  |  | low (2+0) |  | Non-conclusive | R99 | HIV | B24 |  |  | No diagnosis (0+0) | Discrepant |
| **Malignant tumors** | |  |  |  |  |  |  |  |  |  |  |  |  |  |  |  |
|  | **Hepatocellular carcinoma** | |  |  |  |  |  |  |  |  |  |  |  |  |  |  |
| 79 | Hepatocellular carcinoma (HBV) | C22.0 | Viral hepatitis (HBV) | B18.1 |  |  | Very high (4+4) |  | Hepatocellular carcinoma (HBV) | C22.0 | Viral hepatitis (HBV) | B18.1 |  |  | Very high (4+4) | Complete |
| 81 | Hepatocellular carcinoma (HBV) | C22.0 | Viral hepatitis (HBV) | B18.1 |  |  | Very high (4+4) |  | Hepatocellular carcinoma (HBV) | C22.0 | Viral hepatitis (HBV) | B18.1 |  |  | Very high (4+4) | Complete |
| 85 | Hepatocellular carcinoma | C22.0 |  |  |  |  | Very high (4+4) |  | Hepatocellular carcinoma | C22.0 |  |  |  |  | High (4+0) | Complete |
| 92 | Hepatocellular carcinoma (HBV) | C22.0 | Viral hepatitis (HBV) | B18.1 | HIV | B24 | Very high (4+4) |  | Hepatocellular carcinoma (HBV) | C22.0 | Viral hepatitis (HBV) | B18.1 | HIV | B24 | Very high (4+4) | Complete |
| 94 | Hepatocellular carcinoma (HBV) | C22.0 | Viral hepatitis (HBV) | B18.1 |  |  | Very high (4+4) |  | Hepatocellular carcinoma (HBV) | C22.0 | Viral hepatitis (HBV) | B18.1 |  |  | Very high (4+4) | Complete |
|  | **Carcinoma of the uterine cervix** | |  |  |  |  |  |  |  |  |  |  |  |  |  |  |
| 83 | Carcinoma, uterine cervix (HPV16) | C53 | HIV | B21.8 |  |  | Very high (4+4) |  | Non-conclusive | R99 | HIV | B24 |  |  | No diagnosis (0+0) | Discrepant |
| 84 | Carcinoma, uterine cervix (HPV16) | C53 | HIV | B21.8 |  |  | Very high (4+4) |  | Non-conclusive | R99 | HIV | B21.8 |  |  | No diagnosis (0+0) | Discrepant |
| 88 | Carcinoma, uterine cervix (HPV35) | C53 |  |  |  |  | Very high (4+4) |  | Carcinoma, uterine cervix (HPV35) | C53 |  |  |  |  | High (4+0) | Complete |
|  | **Malignant lymphoma** | |  |  |  |  |  |  |  |  |  |  |  |  |  |  |
| 82 | Large B cell lymphoma | C83.3 | HIV | B21.2 |  |  | High (4+0) |  | Large B cell lymphoma | C83.3 | HIV | B21.2 |  |  | High (4+0) | Complete |
| 86 | Large B cell lymphoma | C83.3 | HIV | B21.2 |  |  | High (4+0) |  | Large B cell lymphoma | C83.3 | HIV | B21.2 |  |  | High (4+0) | Complete |
| 91 | Large B cell lymphoma, plasmablastic | C83.3 | HIV | B21.2 |  |  | High (4+0) |  | Large B cell lymphoma, plasmablastic | C83.3 | HIV | B21.2 |  |  | High (4+0) | Complete |
|  | **Kaposi’s sarcoma** | |  |  |  |  |  |  |  |  |  |  |  |  |  |  |
| 80 | Disseminated Kaposi’s sarcoma (HVS-8) | C46.8 | HIV | B21.0 |  |  | Very high (4+4) |  | Disseminated Kaposi’s sarcoma (HVS-8) | C46.8 | HIV | B21.0 |  |  | Very high (4+4) | Complete |
| 90 | Disseminated Kaposi’s sarcoma (HVS-8) | C46.8 | HIV | B21.0 |  |  | Very high (4+4) |  | Disseminated Kaposi’s sarcoma (HVS-8) | C46.8 | HIV | B21.0 |  |  | High (4+0) | Complete |
|  | **Other tumors** | |  |  |  |  |  |  |  |  |  |  |  |  |  |  |
| 87 | Acute myeloid leukemia | C92 | HIV | B21.2 |  |  | High (4+0) |  | Acute myeloid leukemia | C92 | HIV | B21.2 |  |  | High (4+0) | Complete |
| 89 | Undifferentiated malignant neoplasm | C80 | HIV | B21.9 |  |  | High (4+0) |  | Undifferentiated malignant neoplasm | C80 | HIV | B21.9 |  |  | High (4+0) | Complete |
| 93 | Meningothelial meningioma | C71 |  |  |  |  | High (4+0) |  | Non-conclusive | R99 |  |  |  |  | No diagnosis (0+0) | Discrepant |
| **Other diseases** | |  |  |  |  |  |  |  |  |  |  |  |  |  |  |  |
|  | **Complications of cardiovascular diseases** | | |  |  |  |  |  |  |  |  |  |  |  |  |  |
| 95 | Cerebral infarction | I63 | Hypertension | I10 |  |  | High (4+0) |  | Cerebral infarction | I63 |  |  |  |  | Moderate (3+0) | Complete |
| 96 | Diabetic ketoacidosis | E11.1 | Diabetes Mellitus 2 | E11 | Hypertension | I10 | High (4+0) |  | Suggestive of cardiovascular disease | I51.6 |  |  |  |  | Low (2+0) | Discrepant |
| 97 | Cerebral hemorrhage | I61 | Hypertension | I10 |  |  | High (4+0) |  | Suggestive of cardiovascular disease | I51.6 |  |  |  |  | Low (2+0) | Partial |
| 98 | Cerebral infarction | I63 | Hypertension | I10 |  |  | High (4+0) |  | Suggestive of cardiovascular disease | I51.6 |  |  |  |  | Low (2+0) | Partial |
| 101 | Acute myocardial infarction | I21 | Hypertension | I10 | HIV | B24 | High (4+0) |  | Suggestive of cardiovascular disease | I51.6 |  |  | HIV | B24 | Low (2+0) | Partial |
| 103 | Dilated cardiomyopathy | I42.0 | Hypertensive heart and renal disease | I13.9 | HIV | B24 | Moderate (3+0) |  | Suggestive of cardiovascular disease | I51.6 |  |  | HIV | B24 | Low (2+0) | Partial |
| 104 | Hypertensive renal disease with renal failure | I12.0 | Hypertension | I10 |  |  | High (4+0) |  | Suggestive of cardiovascular disease | I51.6 |  |  |  |  | Low (2+0) | Partial |
| 105 | Cerebral hemorrhage | I61 | Hypertension | I10 | HIV | B24 | High (4+0) |  | Non-conclusive | R99 | HIV | B24 |  |  | No diagnosis (0+0) | Discrepant |
| 106 | Diabetic ketoacidosis | E11.1 | Diabetes Mellitus 2 | E11 |  |  | Moderate (3+0) |  | Pneumonia (Group B streptococcus) | J15.3 |  |  |  |  | Low (2+2) | Discrepant |
| 107 | Cerebral hemorrhage | I61 | Hypertension | I10 | HIV | B24 | High (4+0) |  | Non-conclusive | R99 | HIV | B24 |  |  | No diagnosis (0+0) | Discrepant |
| 110 | Cardiac arrest, sudden cardiac death | I46 | Hypertensive heart disease with acute pulmonary hemorrhage | I11 |  |  | Low (2+0) |  | Suggestive of cardiovascular disease | I51.6 |  |  |  |  | Low (1+0) | Partial |
|  | **Pulmonary disease** | |  |  |  |  |  |  |  |  |  |  |  |  |  |  |
| 100 | Pneumoconiosis | J61 |  |  |  |  | Moderate (3+0) |  | Pneumoconiosis | J61 |  |  |  |  | Moderate (3+0) | Complete |
| 109 | Pulmonary fibrosis with pulmonary hypertension | J84 | Asthma | J45 | Pulmonary scars secondary to tuberculosis | B90 | High (4+0) |  | Pulmonary fibrosis | J84 |  |  |  |  | Low (2+0) | Complete |
|  | **Gastrointestinal and Renal disease** | |  |  |  |  |  |  |  |  |  |  |  |  |  |  |
| 99 | Gastric ulcer with upper gastrointestinal hemorrhage | K25.0 | Hypertensive heart disease with (congestive) heart failure | I11.0 |  |  | Moderate (3+0) |  | Suggestive of cardiovascular disease | I51.6 |  |  |  |  | Low (1+0) | Discrepant |
| 102 | Thrombotic microangiopathy with renal necrosis | M31.1 |  |  |  |  | High (4+0) |  | Non-conclusive | R99 |  |  |  |  | No diagnosis (0+0) | Discrepant |
| 108 | Esophagus varices bleeding | I85.0 | Alcoholic fibrosis and sclerosis of liver | K70.2 |  |  | High (4+0) |  | Alcoholic fibrosis and sclerosis of liver | K70.2 |  |  |  |  | Moderate (3+0) | Discrepant |
