# Supplementary material for: Validity of a Minimally Invasive Autopsy for Cause of Death Determination in Adults in Mozambique: An Observational Study
Source: PLoS Med. 2016 Nov 22;13(11):e1002171. doi: 10.1371/journal.pmed.1002171 (PMC5119723; doi:10.1371/journal.pmed.1002171)
Supplement: S2 Text — (DOCX) [file pmed.1002171.s003.docx]

**Supplementary information - Prospective analysis plan**

**Pathological procedures:**

*Minimally invasive autopsy (MIA)*

MIAs will include a detailed external macroscopic examination and the targeted sampling of key-organ tissues by automatic needles (14-18 gauge). Different tissue specimens will be collected from each organ targeted, some samples being fixed in 10% buffered neutral formalin and others directly snap-frozen in liquid nitrogen. Material fixed in formalin will be embedded in paraffin wax and later stained with hematoxylin and eosin. Minimally invasive sampling techniques of proven utility for the diagnosis of malaria (sampling for central nervous system tissue) will be included. Samples will be obtained with the guidance of a portable ultrasound machine, unless blind sampling is shown to be adequate.

There is very limited information in the literature about the optimal number of biopsies required for diagnosis in the MIA approach. However, it seems reasonable to hypothesize that the larger the number of samples the higher the diagnostic accuracy of the procedure.

Samples for microbiological analysis will be obtained under sterile conditions. Consequently, only one biopsy specimen will be obtained with each instrument, and a new instrument will be required for each microbiological sample of each new organ. This imply that at least five disposable automatic biopsy needles will be needed per case (one for the central nervous system, one for each lung, one for the liver, one for the spleen). Samples for blood or CSF culture will be collected with more conventional (and cheaper) needles. Nevertheless, the megacore automatic biopsy instruments used in the study are reusable, which means that multiple guided core biopsies will be obtained with a single instrument. This strategy of multiple biopsies will be used to obtain several biopsy specimens for histological analysis after the initial sample for microbiology has been taken. These new biopsies will be adequate for histological processing and analysis because they do not require sterile conditions.

The number of samples to be taken per case for histological analysis will be: 6 samples from each lung (upper, middle and lower lobes/fields), 2 from the heart, 2 from the spleen, 3 from the liver, 2 from the central nervous system and two samples from the kidney. Finally, additional samples will be obtained from lesions detected in the ultrasound evaluation, or from organs such as the placenta or thymus should those be available.

*Complete diagnostic autopsy (CDA)*

Full post-mortem examination will be done as per the prevailing regulations and procedures to discern underlying, immediate and contributing causes of death based on gross and microscopic findings. CDAs will include a complete dissection of the body and a detailed macroscopic evaluation of each organ using a standardized protocol. Samples will be collected for histological study from all macroscopically identified lesions and from specific organs. Peripheral blood (20 mL) from the inferior vena cava will be collected. In the case of perinatal and neonatal deaths, samples from the umbilical cord and placenta will also be collected. As for MIAs, two tissue specimens will be collected from each organ (formalin-fixed and snap-frozen). If necessary, further histochemical, immunohistochemical (on formalin-fixed, paraffin embedded tissue) or molecular analyses will be performed. Following the procedure the entry points will be closed with purse string sutures.

Standardized pathology procedures, both for the MIA and the CDA, will be predefined after the initial investigators meeting and the standard operating procedures (SOPs) written. Two pathologists will establish the final MIA diagnoses after independently reviewing the histological slides and laboratory results. Similarly, two pathologists will establish the final CDA diagnoses after independent review of the histological slides, clinical records, laboratory results and macroscopic protocols. MIA sampling will be performed first and interpretation will be conducted in a blind manner regarding the CDA findings. After a diagnosis has been reached with the different methods, the rate of MIA-CDA discrepancies will be determined. In addition, the clinical diagnoses obtained from those listed by the clinician on the clinical record will be compared to the autopsy diagnoses, and the rate of clinico-pathological discrepancies on the CoD will be established.

**Microbiological procedures:**

Thorough microbiological investigation of the samples (body fluids and tissues) obtained from the deceased individuals recruited to both the validation and the multicenter study, will allow comprehensive screening of the most frequent infectious diseases in the area, including viruses, bacterias, fungi and parasites. These analyses will complement the information provided by the histological findings. The methodologies used will include classical microbiology procedures and the use of a combination of molecular multiplex techniques, performed in a centralized manner at the Microbiology Department of the *Hospital Clínic*, in Barcelona, Spain. However, an important limitation of the microbiological examination of postmortem samples is that postmortem culture isolates may contain mixed bacterial flora, some of which may be difficult to grow using standard culture methods, or may be overgrown by other bacteria. Molecular techniques will allow the detection of more fastidious organisms, and in combination with control samples (such as the samples from the contralateral lung) will allow differentiation from background postmortem non-pathogenic contaminants. All the individuals recruited with clinical or pathological evidence of infection will be analyzed, starting with conventional cultures and followed, if necessary, by more specific and sensitive molecular techniques. A systematic PCR screening for all potential microorganisms related to death is not currently feasible due to the large number of microorganisms and cost of the molecular techniques. Therefore, before study initiation, a logical algorithm will be predefined, that will include the patient’s age, the pathology lesions, the classical microbiology and parasitology data, and complemented, if necessary, by more specific and sensitive molecular techniques. Thus, for some patients, it will not be necessary to perform pathogen-specific techniques should the cause of death be clearly identified by the pathology examinations and classical microbiology. Contrarily, some patients may need a wide variety of pathogen specific and/or generic PCRs to investigate other causes of death. Detailed and standardized microbiological procedures and priority algorithms will be predefined after the initial workshop meeting and included in the SOPs.

*Examples of the microbiology procedures:*

*Procedure 1. Classical microbiological culture of samples and parasitological investigations*

Postmortem microbiology cultures have long been said to be of little value. Indeed, microbiological contamination of postmortem tissue and lack of detection sensitivity secondary to the fastidiousness of a pathogen or the diagnostic test chosen to detect it are major potential limitations to applying classical microbiology techniques to postmortem samples. Thus, classical culture methods may be of great use to enhance diagnostic capacities when trying to ascertain likely causes of death, and a first step to perform in the diagnosis of infections. Correct interpretation of the microbiological results must be taken into consideration: i. the sample from which the microorganism has been isolated; ii. potential pathogenicity of the isolated microorganism and iii. the habitual microbiota of the sample. Direct inoculation of other samples such as body fluid or tissues will be carried out using conventional methods for isolation of aerobes and anaerobes: blood agar, chocolate agar, MacConkey and colistin-nalidixic acid agar. Schaedler agar will be used to isolate anaerobic bacteria. In addition, broth such as, thioglycolate will be inoculated. Thayer-Martin agar will be inoculated from CSF, tissues or respiratory samples when meningococcemia, bacterial meningitis or sepsis is suspected. All human tissues will be ideally removed no later than 4–6 hours postmortem (more flexibility will be allowed in deaths occurring in the community) and processed as follows: the tissue sample will be added into a glass-teflon homogenizer with 1 ml of sterile saline solution and gently homogenized with several strokes of the piston up to full homogenization of the tissue. Part of the homogenized sample will then be inoculated on the abovementioned media. The remaining homogenised sample will be kept frozen at -80ºC in free DNAasa/Rnase tubes for further analysis by molecular tools. Classical microscopy will also be used to investigate malarial infection, and PCR of tissues will be performed to confirm *P. falciparum* parasites in different tissues. In cases in where no precise etiological diagnosis has been made by classical microbiology, nucleic acids will need to be extracted and more sensitive molecular techniques will be applied.

*Procedure 2. Samples processing and nucleic acid extraction*

We will analyze whole blood, plasma, cerebrospinal fluid (CSF), amniotic fluid (if available), other available sterile body fluids and homogenized tissue samples. Whole blood will be collected in tubes containing EDTA. The isolation of DNA from directly frozen tissues (preferably) or after long-term storage of paraffin-preserved samples will be performed following standard procedures. This protocol will initially be re-evaluated for isolation of viral RNA/DNA and bacterial DNA. Protocols will be developed for transporting samples to the Microbiology Department of the Hospital Clinic in Barcelona where there are high throughput extraction facilities to process these samples.

*Procedure 3. Application of broad spectrum PCR for detection of bacteria and fungal infections.*

Total DNA/RNA from blood will be extracted followed by a procedure to reduce background interference from the host DNA. The amplification and further sequencing of the 16S rRNA gene will be used to detect the bacteria causing the infection. The sequence obtained will be analyzed performing a blast with two different databases. Cultures of the samples will be carried out in specific culture media according to the PCR 16S rRNA results. The isolation of specific bacteria will detect antimicrobial susceptibility. Special attention will be given to detect certain specific pathogens (such as *N. meningitides*, *S. pneumonia*, *M. tuberculosis*, *T. gondii,* cytomegalovirus, etc.) and, if needed, specific PCR methods will be used. Moreover, a panfungal PCR assay will be used based on the specific amplification and sequencing of the complete internal transcribed spacer (ITS) 1 and 2 regions of the ribosomal DNA complex.

*Procedure 4. Detection of viral infections*.

Detection of viral infections will be performed by serological and molecular methods. Infections by human immunodeficiency virus (HIV), hepatitis B (HBV) and hepatitis C (HCV) will be screened by detection of antibodies (HIV, HCV) or antigen (HBV), followed by quantification of the viral load if needed. Other viral infections will be investigated by serological or molecular techniques based on the clinical information available and pathological findings. For example: i) generic RT-PCR techniques will be used to detect members of the *Flavivirus* genus such as dengue and yellow fever and members of the *Arenavirus* genus such as *Lymphocytic choriomeningitis virus*; ii) amultiplex PCR will be used to detect a variety of respiratory viruses including influenza and respiratory syncitial virus, as well as enteroviruses; iii) Herpes group viruses such as Cytomegalovirus, Epstein Barr, Herpes Simplex or Varicella zoster viruses will be detected by specific real time PCR methods.
